# Mechanistic characterization of disulfide bond reduction of an ERAD substrate mediated by cooperation between ERdj5 and BiP

**DOI:** 10.1016/j.jbc.2023.105274

**Published:** 2023-09-21

**Authors:** Xiaohan Cai, Shogo Ito, Kentaro Noi, Michio Inoue, Ryo Ushioda, Yukinari Kato, Kazuhiro Nagata, Kenji Inaba

**Affiliations:** 1 Institute of Multidisciplinary Research for Advanced Materials, Tohoku University, Sendai, Miyagi, Japan; 2 Department of Molecular and Chemical Life Sciences, Graduate School of Life Sciences, Tohoku University, Sendai, Miyagi, Japan; 3 Department of Biotechnology and Life Science, Tokyo University of Agriculture and Technology, Koganei, Tokyo, Japan; 4 Faculty of Life Sciences, Kyoto Sangyo University, Kyoto, Japan; 5 Graduate School of Medicine, Tohoku University, Sendai, Japan; 6 Department of Chemistry, Graduate School of Science, Tohoku University, Sendai, Miyagi, Japan; 7 Medical Institute of Bioregulation, Kyushu University, Fukuoka, Japan; 8 Core Research for Evolutional Science and Technology (CREST), Japan Agency for Medical Research and Development (AMED), Tokyo, Japan

**Keywords:** ERdj5, ER-associated degradation, BiP, high-speed AFM, J-chain, ER proteostasis

## Abstract

Endoplasmic reticulum (ER)-associated degradation (ERAD) is a protein quality control process that eliminates misfolded proteins from the ER. DnaJ homolog subfamily C member 10 (ERdj5) is a protein disulfide isomerase family member that accelerates ERAD by reducing disulfide bonds of aberrant proteins with the help of an ER-resident chaperone BiP. However, the detailed mechanisms by which ERdj5 acts in concert with BiP are poorly understood. In this study, we reconstituted an *in vitro* system that monitors ERdj5-mediated reduction of disulfide-linked J-chain oligomers, known to be physiological ERAD substrates. Biochemical analyses using purified proteins revealed that J-chain oligomers were reduced to monomers by ERdj5 in a stepwise manner *via* trimeric and dimeric intermediates, and BiP synergistically enhanced this action in an ATP-dependent manner. Single-molecule observations of ERdj5-catalyzed J-chain disaggregation using high-speed atomic force microscopy, demonstrated the stochastic release of small J-chain oligomers through repeated actions of ERdj5 on peripheral and flexible regions of large J-chain aggregates. Using systematic mutational analyses, ERAD substrate disaggregation mediated by ERdj5 and BiP was dissected at the molecular level.

Secretory proteins, translocated across the endoplasmic reticulum (ER) membrane in an unfolded form (1), fold with the help of ER-resident molecular chaperones, protein disulfide isomerase family members, N-glycosylation enzymes, and various other proteinaceous and chemical factors. Stringent cellular quality control systems allow only correctly folded proteins to exit the ER for transport to their final destinations (2). By contrast, misfolded proteins tend to accumulate in the ER and induce severe ER stresses, resulting in harmful effects on living organisms. This can result in a variety of diseases such as Alzheimer’s disease (3), Parkinson’s disease (4), and type-2 diabetes (5). To maintain ER proteostasis, misfolded proteins are retrogradely delivered from the ER to the cytosol to be degraded by proteasomes in a process called ER-associated degradation (ERAD) (6, 7). During this process, disulfide bond reduction of misfolded proteins is likely a critical step that facilitates their passing through the dislocon channel, thereby promoting their ERAD without pausing (8).

DnaJ homolog subfamily C member 10 (ERdj5) was discovered as a protein disulfide isomerase family member that catalyzes disulfide reduction of misfolded proteins in the oxidative environment of the ER and accelerates their ERAD (9, 10). Misfolded “glycoproteins” are first recognized by ER degradation–enhancing mannosidase-like protein 1 (11) and reduced by ERdj5 *via* recruitment of ERdj5 by ER degradation–enhancing mannosidase-like protein 1 (12). The reduced proteins are then delivered to the dislocon channel (Sec61) by immunoglobulin-binding protein (BiP), translocated to the cytoplasm, and eventually degraded by the ubiquitin-proteasome system (13). On the other hand, misfolded “non-glycoproteins” are first recognized by BiP and then transferred to ERdj5 *via* specific interactions between the ERdj5 J-domain and BiP (14). In either case, the cooperation of ERdj5 and BiP is believed to be essential for enhancing ERAD.

Our previous crystallographic studies revealed that ERdj5 consists of N-terminal and C-terminal clusters and that these two are connected by a flexible linker loop (10, 15). In total, there are six tandem thioredoxin (Trx)-like domains in ERdj5, four (Trx1−4 domain) of which have a CXXC motif at the redox-active site. While the N-terminal cluster contains Trx1, Trx2, and two redox-inactive Trx domains, the C-terminal cluster is composed of Trx3 and Trx4 domains. The redox potential of each Trx-like domain has been determined to be −166 mV for Trx1 domain, −172 mV for Trx3 domain, and −185 mV for Trx4 domain. Notably, the CXXC motif of Trx2 domain is masked by the J-domain, preventing substrate molecules from accessing this redox-active site. As a consequence, the reducing ability of Trx2 domain is much weaker than that of the other Trx-like domains. Mutational analyses further demonstrated that Trx3 domain and Trx4 domain in the C-terminal cluster are the primary reductase domains responsible for the ERAD-enhancing activity of ERdj5 (10), while the N-terminal J-domain with a His-Pro-Asp (HPD) motif is essential for binding BiP and stimulating its ATPase activity (16). More recently, another crystal structure was determined for ERdj5, in which the relative orientation and location of the C-terminal cluster to the N-terminal one differ largely from those in the original structure (15). High-speed atomic force microscopy (HS-AFM) measurements revealed that these two different conformations are in equilibrium, concomitant with the rapid movement of the C-terminal cluster (15).

While physiological and biochemical studies have also since been conducted to investigate roles of ERdj5 as a disulfide reductase (17, 18, 19), detailed mechanisms of the ERdj5-BiP interplay during the disulfide reduction of ERAD substrates remains poorly understood. In this study, we set up an *in vitro* experimental system that monitors J-chain oligomer disaggregation mediated by the cooperation of ERdj5 and BiP. J-chain, a protein component of immunoglobulins IgM and IgA (20), contains eight cysteine residues and has a molecular mass of ∼25 kDa per monomer. It forms disulfide-linked oligomers when overexpressed alone in cells, and the oligomers undergo ERAD in an ERdj5-dependent manner (9, 15). The present *in vitro* analysis using purified proteins enabled us to follow the kinetics of J-chain oligomer disassembly mediated by ERdj5 and BiP and demonstrated that BiP synergistically accelerates ERdj5-catalyzed disulfide reduction *via* generation of trimeric and dimeric intermediate species. In support of this, real-time, single-molecule observations using HS-AFM demonstrated that small J-chain fragments were released from the large J-chain oligomers through repeated attacks of ERdj5 on peripheral and flexible regions of the oligomers. Systematic mutational analyses provided further mechanistic insights into ERAD substrate reduction mediated by the cooperation of ERdj5 and BiP.

## Results

### J-chain reduction mediated by ERdj5 and/or BiP

**Figure 1:**
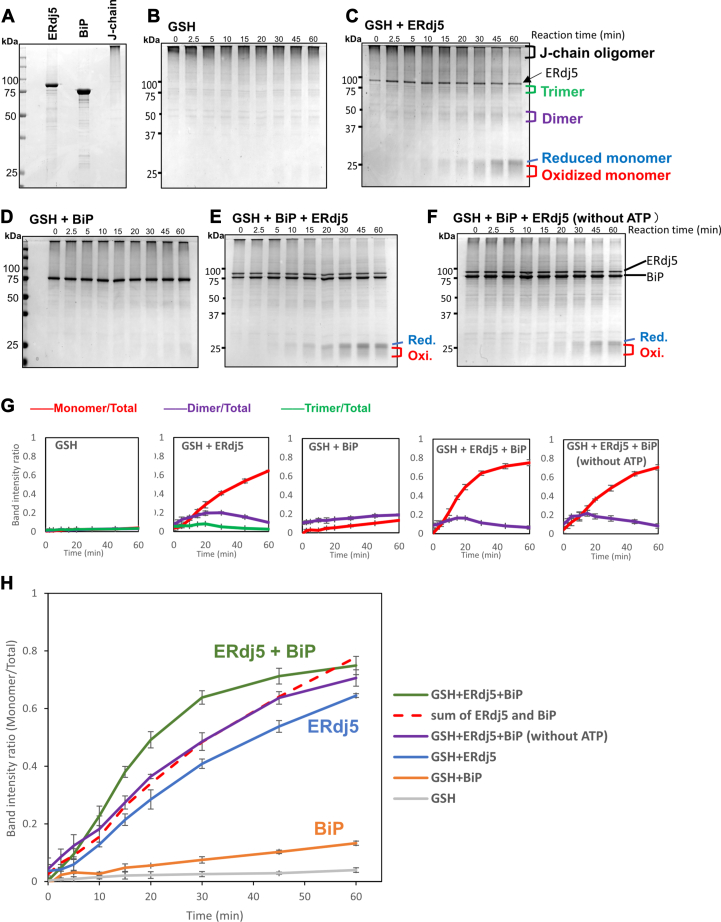
**J-chain reduction mediated by ERdj5 and/or BiP.***A*, purified ERdj5, BiP, and J-chain oligomer. Proteins were separated by nonreducing SDS-PAGE and stained with CBB-G250. *B*–*F*, 5 μM J-chain oligomers were reacted with 2.5 mM GSH, 0.5 μM ERdj5, and/or 0.5 μM BiP in the presence of 1 mM ATP (without ATP in *F*) at 30 °C for the specified time. Reactions were quenched with NEM at the indicated timepoints, and 4 μl of the reaction solution was subjected to nonreducing SDS-PAGE and stained with CBB G-250. *G*, band intensity ratio of J-chain monomers (*red*), dimers (*magenta*), and trimers (*green*) *versus* total J-chain on each nonreducing SDS-PAGE gel was plotted as a function of reaction time. Results are means ± SD of three independent experiments. *H*, band intensity ratio of J-chain monomer *versus* total J-chain on nonreducing SDS-PAGE gels plotted as a function of reaction time based on the results shown in (*B*–*F*). Bip, binding protein; CBB, Coomassie brilliant blue; NEM, N-ethylmaleimide.

To analyze the ERdj5-mediated disulfide bond reduction of an ERAD substrate, we reconstituted the J-chain oligomer reduction system using nonreducing SDS gels and purified ERdj5, BiP, and J-chain oligomers (Fig. 1*A*). GSH was added as a source of reducing power for ERdj5 (21). Given that GSH itself may have the potential to reduce J-chain oligomers, and that GSH is abundant in the ER, ranging from 1 to 10 mM (22), we first verified that 2.5, 5, and 10 mM GSH hardly reduced J-chain oligomers even after 60 min of reaction time (Fig. S1*A*). To minimize the effect of GSH itself and assess the net contribution of ERdj5 in J-chain reduction, we chose 2.5 mM GSH for this assay system. The reaction was initiated by adding purified ERdj5 (0.5 μM) and/or BiP (0.5 μM) to J-chain oligomer (5 μM; 10-fold excess to enzymes) solution in the presence of 2.5 mM GSH and 1 mM ATP and was stopped by addition of 10 mM N-ethylmaleimide (NEM) at different timepoints. The human BiP protein used in this study retained ATPase activity, which was enhanced by ERdj5 (Fig. S2).

Whereas GSH alone hardly reduced J-chain oligomers (Fig. 1*B*), ERdj5 significantly promoted their reduction to monomers (Fig. 1*C*). Importantly, a small amount of trimer and larger amount of dimer were generated during the early stages of J-chain reduction and thereafter both decreased gradually (at 45 and 60 min). Meanwhile, monomeric J-chain was greatly increased in the latter stages of the reaction, suggesting the stepwise reduction and conversion of J-chain oligomers to monomers *via* trimer and dimer species (Fig. 1, *C* and *G*). The monomer species consisted of oxidized and reduced forms, with the reduced form predominating (Fig. 1*C*), suggesting that ERdj5 could reduce both intermolecular and intramolecular disulfide bonds of J-chains. In the reducing SDS-PAGE, the J-chain molecules were all converted to the dimer and monomer species (Fig. S1*B*). Notably, the sequential reduction of the PA-tagged J-chain oligomers to the trimer, dimer, and monomer was further verified by the Western blotting analysis using an anti-PA antibody (Fig. S3). However, given that different oligomeric states of proteins may show different membrane transfer efficiencies and/or different reactivity with an antibody used, we used the Coomassie brilliant blue (CBB)-staining data for the quantitative analysis throughout this work.

BiP itself does not contain redox-active sites, hence no monomer species were generated upon addition of BiP without GSH (Fig. S1*C*). However, BiP only generated a small amount of J-chain dimers and monomers in the presence of 2.5 mM GSH (Fig. 1, *D* and *G*). Notably, when both ERdj5 and BiP were added to the reaction solution, J-chain oligomers were reduced to monomers more efficiently than expected from the simple sum of the activities of ERdj5 and BiP (Fig. 1, *E*, *G*, and *H*), indicating that they act synergistically to accomplish efficient J-chain reduction. No synergy was observed in the absence of ATP (Fig. 1, *F*–*H*), suggesting that the ATPase activity of BiP is required for this action.

### Mechanisms of BiP-dependent acceleration in ERdj5-catalyzed J-chain reduction

**Figure 2:**
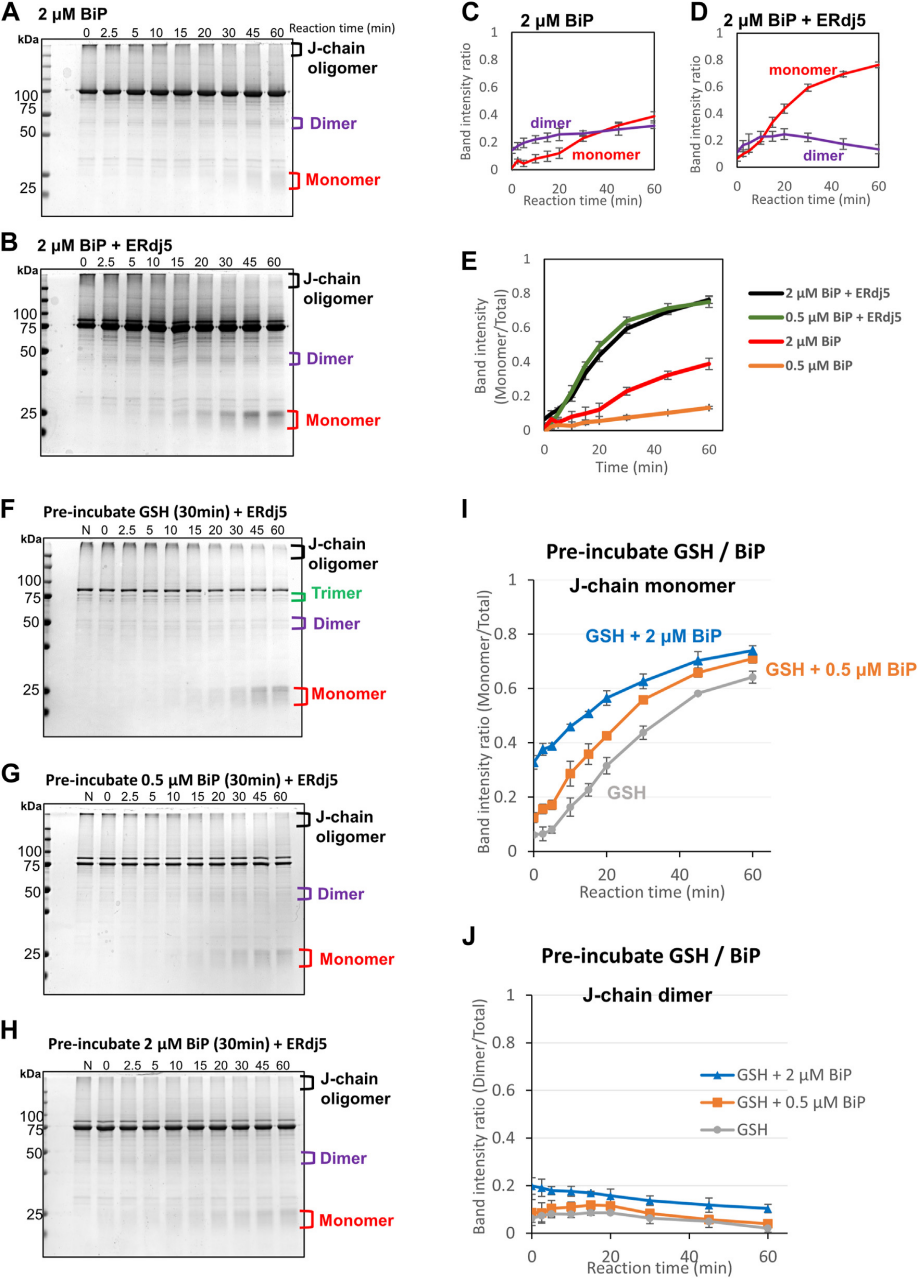
**Effects of different concentrations of BiP and their preincubation on ERdj5-mediated J-chain reduction.***A* and *B*, 5 μM J-chain oligomers were reacted with 2 μM BiP, 2.5 mM GSH, and 1 mM ATP in the absence or presence of 0.5 μM ERdj5 for the indicated time at 30 °C. Reaction solutions quenched with NEM at the indicated timepoints were subjected to nonreducing SDS-PAGE and stained with CBB G-250. *C* and *D*, band intensity ratio of J-chain monomers (*red*) and dimers (*magenta*) *versus* total J-chain on each nonreducing SDS-PAGE gel was plotted as a function of reaction time based on the results shown in (*A* and *B*). *E*, band intensity ratio of J-chain monomers *versus* total J-chain on each nonreducing SDS-PAGE gel was plotted as a function of reaction time based on the results shown in (*A*, *B*, and Fig. 1, *D* and *E*, and *F*–*H*). 5 μM J-chain oligomers were preincubated with 0, 0.5, or 2 μM BiP for 30 min before being mixed with 0.5 μM ERdj5 and 2.5 mM GSH in the presence of 1 mM ATP for the indicated time at 30 °C. Reaction solutions quenched with NEM at the indicated timepoints were subjected to nonreducing SDS-PAGE and stained with CBB G-250. Lane N, no preincubation with GSH, and BiP. *I* and *J*, band intensity ratio of J-chain monomer *versus* total J-chain (*I*) and dimer *versus* total J-chain (*J*) on nonreducing SDS-PAGE gels plotted as a function of reaction time. Results are means ± SD of three independent experiments. Bip, binding protein; CBB, Coomassie brilliant blue; NEM, N-ethylmaleimide.

BiP is abundant in the ER as a primary chaperone that can respond to ER stress (23). To investigate the role of BiP in disaggregation of J-chain oligomers, we used a higher concentration (2 μM) of BiP in the J-chain reduction assay. At 2 μM, BiP enhanced the generation of J-chain dimers and monomers with GSH (Fig. 2*A*); after 60 min, ∼3-fold more monomer was generated by 2 μM BiP than by 0.5 μM BiP (Fig. 2*E*). In the presence of 0.5 μM ERdj5 and 2 μM BiP, J-chain oligomers were converted to monomers much more rapidly (Fig. 2, *B* and *D*). However, the efficiency was almost the same as that of the combination of 0.5 μM ERdj5 and 0.5 μM BiP (Fig. 2*E*), indicating that excess BiP was unable to enhance further the reduction of J-chain oligomers. This result suggests that stoichiometric interplay between ERdj5 and BiP enables their synergistic actions for J-chain reduction in which the BiP-recruited substrate can be efficiently transferred to and reduced by ERdj5 *via* specific interactions between BiP and ERdj5. In this case, residual BiP would function as a molecular chaperone to disaggregate J-chain oligomers, but this effect seems marginal compared with the cooperative effect of ERdj5 and BiP.

Nonetheless, to assess the function of BiP as a molecular chaperone that promotes disaggregation, we explored the effect of BiP preincubation on ERdj5-mediated J-chain reduction. To this end, J-chain oligomers were preincubated with 0.5 or 2 μM BiP for 30 min and then reacted with 0.5 μM ERdj5 (Fig. 2, *F*–*J*). In this case, preincubation with BiP greatly increased the amount of monomer and dimer species, especially at early timepoints of the reaction (0–20 min) and in a manner dependent on the BiP concentration (Fig. 2, *G*–*J*). While preincubation with 0.5 μM BiP yielded monomer species representing ∼40% of the total after 20 min, the yield was increased to ∼55% of the total following preincubation with 2 μM BiP (Fig. 2*I*). Thus, BiP can serve as an effective facilitator of the subsequent ERdj5-mediated disulfide reduction. BiP performs dual roles, acting as general chaperone that disaggregates aberrant oligomers, and as a specific partner that recruits substrates to ERdj5, and its role likely changes depending on the stage at which it participates in the ERdj5-dependent ERAD pathway.

**Figure 3:**
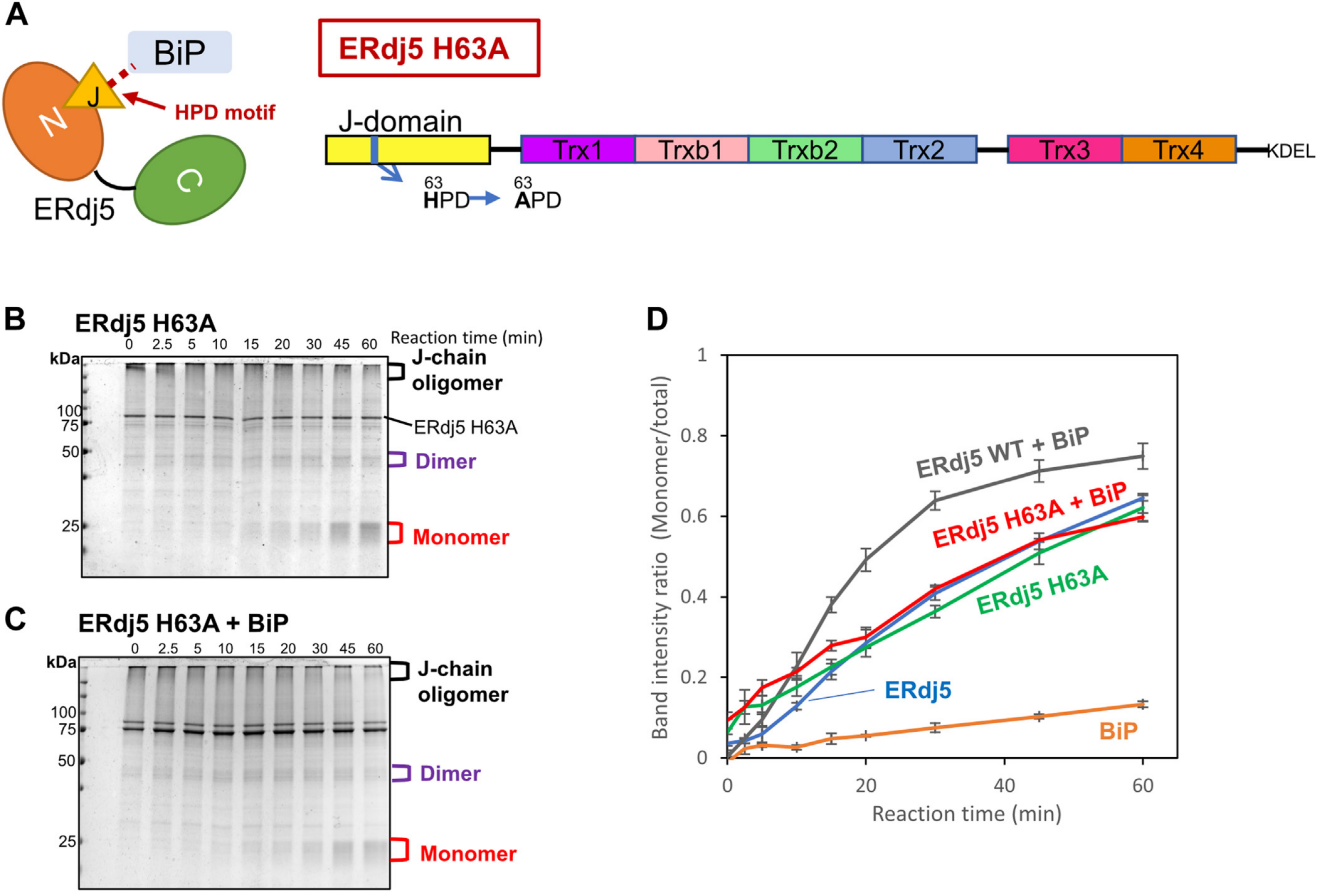
**J-chain reduction mediated by the ERdj5 J-domain mutant.***A*, simplified cartoon representing the ERdj5–BiP interaction through the HPD motif of the ERdj5 J-domain and the domain organization of ERdj5 H63A (*B* and *C*), 5 μM J-chain oligomers were reacted with 0.5 μM ERdj5 H63A, 2.5 mM GSH, and 1 mM ATP in the absence or presence of 0.5 μM BiP for the indicated time at 30 °C. Reaction solutions quenched with NEM at the indicated timepoints were subjected to nonreducing SDS-PAGE and stained with CBB G-250. *D*, band intensity ratio of J-chain monomer *versus* total J-chain on nonreducing SDS-PAGE gels plotted as a function of reaction time. Results are means ± SD of three independent experiments. Bip, binding protein; CBB, Coomassie brilliant blue; HPD, His-Pro-Asp; NEM, N-ethylmaleimide.

To further verify the importance of ERdj5-BiP interplay in J-chain oligomer reduction, we prepared an ERdj5 mutant defective in its interaction with BiP. ERdj5 is known to interact with and activate BiP through the HPD motif located in the J-domain (9). Accordingly, an H63A mutation at this motif is predicted to abolish the ability of ERdj5 to activate BiP. Indeed, the ATPase activity of BiP was enhanced by ERdj5 WT, but not by the H63A mutant (Fig. S2). The J-chain reduction assay revealed that the H63A mutant retained the ability to reduce J-chain oligomers to monomers as efficiently as WT ERdj5 (Fig. 3*B*). Interestingly, however, BiP did not further accelerate J-chain reduction mediated by the ERdj5 H63A mutant (Fig. 3*D*). The increase of a BiP concentration to 2 μM did not enhance the catalysis of ERdj5 H63A (Fig. S4, *A* and *B*), consistent with the observation that this mutant was unable to enhance the BiP ATPase activity at this BiP concentration (Fig. S4*C*). These results indicate the essentiality of the functional interplay between ERdj5 and BiP in accelerated J-chain reduction by ERdj5. In other words, BiP can further promote ERdj5-catalyzed J-chain reduction by forming an activated complex with ERdj5 *via* the HPD motif.

### Distinct roles of ERdj5 Trx-like domains in J-chain reduction

**Figure 4:**
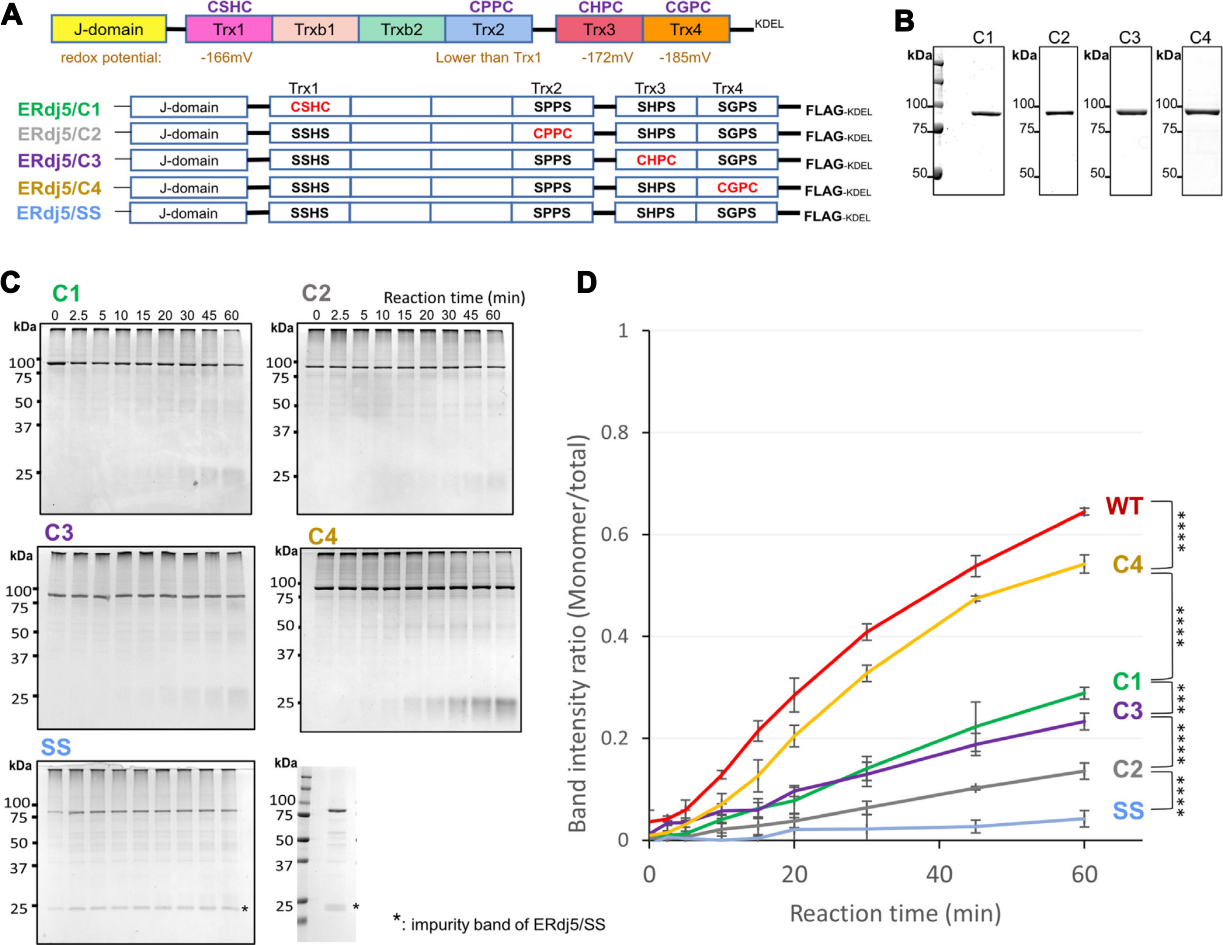
**J-chain reduction mediated by ERdj5 Trx-likedomain mutants.***A*, domain organization of ERdj5 based on the present crystal structure analysis. The CXXC sequences indicate the locations of redox-active sites in Trx domains. *B*, purified ERdj5 Trx-like domain mutants. Proteins were separated by nonreducing SDS-PAGE and stained with CBB-G250. *C*, 5 μM J-chain oligomers were reacted with 2.5 mM GSH and 0.5 μM ERdj5 Trx-domain mutants in the presence of 1 mM ATP for the indicated time at 30 °C. Reaction solutions quenched with NEM at the indicated timepoints were subjected to nonreducing SDS-PAGE and stained with CBB G-250. *D*, band intensity ratio of J-chain monomer *versus* total J-chain on nonreducing SDS-PAGE gels plotted as a function of reaction time in (*C*). Results are means ± SD of three independent experiments. Data were analyzed by one-way ANOVA and Tukey tests. Statistically significant differences between each ERdj5 derivative are indicated by *asterisks* (∗∗∗*p* < 0.001; ∗∗∗∗*p* < 0.0001). CBB, Coomassie brilliant blue; NEM, N-ethylmaleimide; Trx, thioredoxin.

Our previous studies demonstrated that Trx3 domain and Trx4 domain present in the C-terminal cluster are redox-active domains essential for ERAD acceleration in cells (10). To further investigate the roles of Trx3 domain and Trx4 domain in disulfide reduction of misfolded proteins, we prepared a set of ERdj5 mutants (C1−C4) in which only one CXXC motif was retained, while the other three were substituted by SXXS (Fig. 4*A*). After overexpression in HEK293T cells and purification (Fig. 4*B*), the mutant proteins were used in the J-chain reduction assay. The results demonstrated that the ERdj5 C4 mutant was the most efficient in reducing J-chain oligomers to monomers, although its efficiency was slightly lower than that of the WT. C1 and C3 reduced J-chain oligomers to monomers to a lesser extent than C4. C2 displayed the lowest reductase activity, consistent with our structural observation that the redox-active site in Trx2 domain is masked by the N-terminal J-domain of ERdj5 (10).

**Figure 5:**
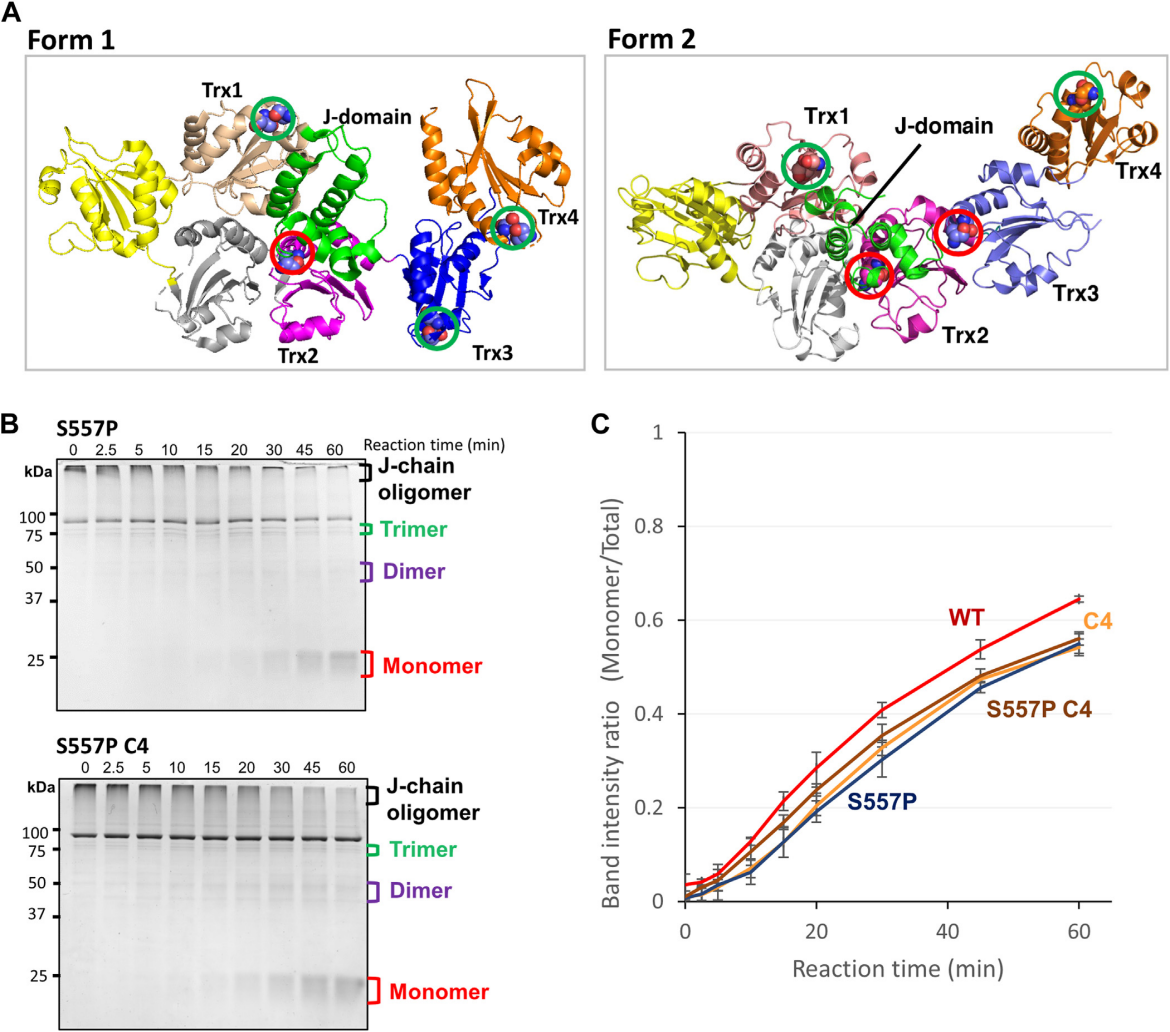
**J-chain reduction mediated by the ERdj5 conformation-fixed mutant.***A*, crystal structure of ERdj5 S557P (form-1, PDB: 5AYK) and ERdj5 CC (form-2, PDB: 5AYL). The CXXC motifs in Trx1, Trx2, Trx3, and Trx4 domains are *circled in green* (exposed to surface) or *red* (concealed). *B*, 5 μM J-chain oligomers were reacted with 2.5 mM GSH and 0.5 μM ERdj5 form-1 mutants in the presence of 1 mM ATP for the indicated time at 30 °C. Reaction solutions quenched with NEM at the indicated timepoints were subjected to nonreducing SDS-PAGE and stained with CBB G-250. *C*, band intensity ratio of J-chain monomer *versus* total J-chain on nonreducing SDS-PAGE gels plotted as a function of reaction time. Results are means ± SD of three independent experiments. CBB, Coomassie brilliant blue; NEM, N-ethylmaleimide; Trx, thioredoxin.

The above results demonstrated that Trx4 domain possesses strong reductase activity against J-chain oligomers (10), whereas Trx3 is inferior as a reductase domain to Trx4. The difference between the reductase activities of Trx4 domain and Trx3 domain could be due to the alternating domain architecture of ERdj5. Our previous crystallographic studies revealed two different ERdj5 conformations with different domain arrangements; form-1 and form-2 (15). In form-1, the CXXC motifs in Trx1, Trx3, and Trx4 domains are exposed to bulk solvent, whereas in form-2, the CXXC motif in Trx3 domain is directed toward the inside of the molecule (Fig. 5*A*). Thus, Trx3 domain seems unlikely to function as an effective reductase domain in form-2.

To explore the importance of the alternating conformations of ERdj5 in efficient J-chain reduction, we prepared the ERdj5 S557P mutant, which was previously shown to adopt form-1 exclusively and constitutively (15). The S557P mutant reduced J-chain oligomers to monomers, but its efficiency was only slightly lower than that of WT ERdj5 (Fig. 5, *B* and *C*). We also constructed and purified the S557P C4 mutant, which retained a redox-active CXXC motif only in Trx4 domain in the S557P mutant. This mutant reduced J-chain oligomers to monomers at almost the same rate as ERdj5 S557P. Thus, Trx4 domain retained high J-chain reduction activity even when ERdj5 was fixed in form-1, suggesting that the reductase activity of Trx4 domain does not rely on the alternating domain arrangements of ERdj5. By contrast, the contribution of the other Trx-like domains seems negligible, as far as ERdj5 maintains a form-1 conformation.

### HS-AFM observation of ERdj5 acting on J-chain oligomers

**Figure 6:**
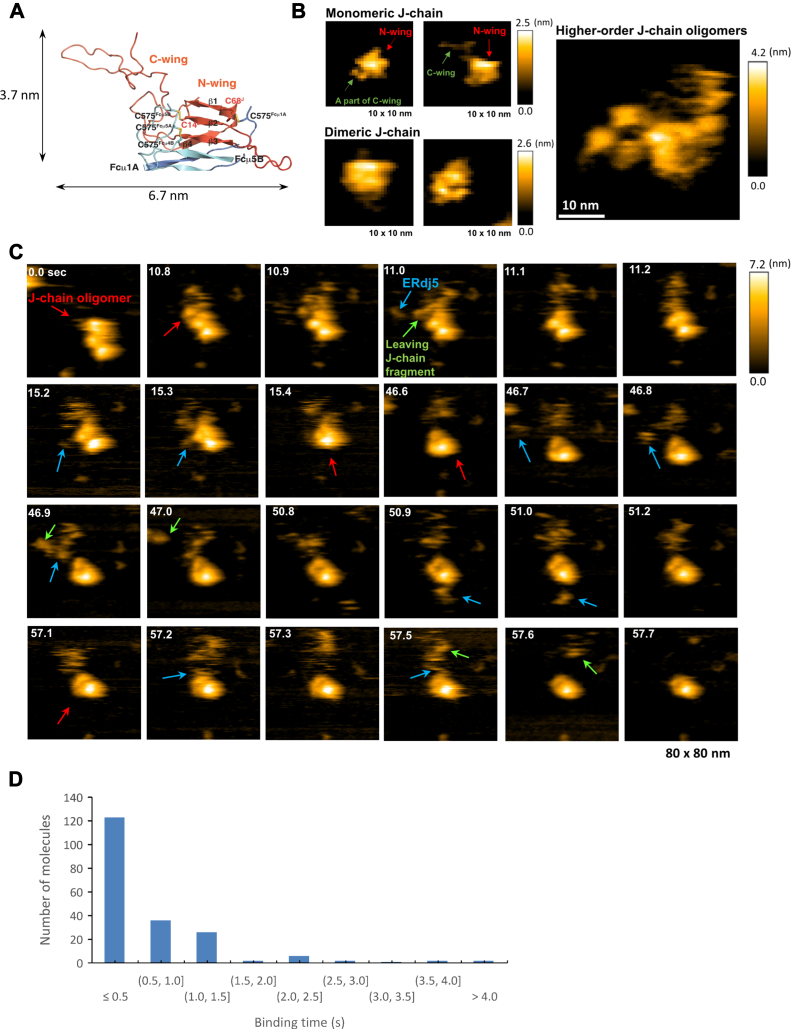
**HS-AFM observation of ERdj5 acting on J-chain oligomers.***A*, structure of the J-chain (monomer) based on cryo-EM analysis of IgM (PDB: 6KXS). Fcμ and J-chain are colored *blue and orange*, respectively. J-chain has a two-winged structure and interacts with the tailpieces of the Fcμ pentamer. *B*, high-speed atomic force microscopy (HS-AFM) images of J-chain molecules differing in size and compactness. 100 nM J-chain was used for immobilization onto the cobalt-scattered mica surface. After 80 min incubation with 2.5 mM GSH, J-chain molecules were mostly observed as higher-order oligomers, while the minor portion as monomers and dimers. *C*, a typical set of HS-AFM snapshots showing that ERdj5 molecules bound to the peripheral regions of the J-chain oligomer, eventually leading to its disaggregation. J-chain oligomers, ERdj5 molecules, and leaving J-chain fragments are indicated by *red, blue, and green arrows*, respectively. *D*, histogram of the binding time between ERdj5 molecules and J-chain oligomers. Samples (n = 200) were randomly selected from particles that were repeatedly attacked by ERdj5 and eventually disaggregated on the mica surface.

To directly visualize the action of ERdj5 on J-chain oligomers at the single-molecule level, we employed HS-AFM (24, 25). For this, target molecules are immobilized onto a mica surface, and their three-dimensional structure and dynamics are analyzed by rapidly scanning the surface using a cantilever arm with an extremely sharp tip. After numerous attempts under various conditions, we optimized the experimental conditions for HS-AFM measurement. Briefly, sample solution containing 100 nM J-chain oligomers and 2.5 mM GSH was loaded onto a cobalt-scattered mica surface. After J-chain molecules were immobilized, the surface was washed to remove unbound J-chain molecules, and images were acquired. Based on the crystal structure of a J-chain monomer (26) (Fig. 6*A*), its N-wing domain is rich in β-strand elements, whereas the C-wing segment is intrinsically disordered. Indeed, our HS-AFM images visualized the N- and C-wings of a J-chain monomer generated as a minor species after an 80 min incubation with 2.5 mM GSH and no ERdj5 (Fig. 6*B*). Based on the overall shape and dimension, J-chain monomers, dimers, and higher-order oligomers were roughly identified using this approach (Fig. 6*B*). Consistent with the above biochemical assay (Fig. 1*B*), most of the J-chain molecules maintained oligomeric states in the presence of 2.5 mM GSH, even after HS-AFM observation. These observations confirm that HS-AFM is a useful tool for following the disassembly of J-chain oligomers during ERdj5-catalyzed disulfide reduction.

Next, we performed HS-AFM observation for the ERdj5-mediated J-chain reduction (Fig. 6*C*). To this end, 10 nM ERdj5 was added to the cantilever holder to initiate the J-chain reduction on the mica surface, and the data acquisition was started immediately. Surface coating with Co^2+^ prevented the adherence of excess ERdj5 molecules. To confirm the overall shapes of ERdj5 molecules and thereby distinguish them from the J-chain molecules, we first performed the HS-AFM measurements for ERdj5 alone. Consistent with previous observations (15, 25), we observed that ERdj5 molecules adopt multiple conformations with different orientations between the N- and C-terminal clusters (Fig. S5).

Figure 6*C* shows a typical set of HS-AFM images, demonstrating the disassembly of J-chain oligomers upon ERdj5 action. Time-resolved snapshots revealed that an ERdj5 molecule attacked a peripheral region of the J-chain oligomer at 11.0 s and thereafter a part of the J-chain oligomer was released at 11.1 s (Fig. 6*C*). Similar events were observed at different regions of the J-chain oligomer at 46.7 s and 57.5 s, which eventually resulted in J-chain oligomers of smaller sizes as seen in snapshots at 47.0 s and 57.7 s, respectively (Fig. 6*C*). We additionally observed that an ERdj5 molecule attacked a core part of the J-chain oligomer from the downside at 50.9 and 51.0 s (Fig. 6*C*). However, no J-chain fragments were released in this case. Collectively, it is suggested that the ERdj5-mediated disassembly of the J-chain oligomers tends to occur when the enzyme attacks peripheral and/or flexible regions of the J-chain oligomers.

To distinguish ERdj5 particles from the J-chain fragments leaving from the complexes with certainty and thereby interpret the HS-AFM data objectively, we calculated the areas and circularities of particles observed in HS-AFM images (25, 27, 28). The area *versus* circularity plot classified randomly chosen ERdj5 particles into two major groups, round O-shape (form-1) and extended V-shape (form-2) (Fig. 6*A* left and Fig. S6*B*), consistent with our previous crystallographic and HS-AFM studies (Figs. 5*A* and S5*A*) (15). The HS-AFM observation of J-chain alone in the presence of GSH visualized its dimer and monomer species located nearby the higher-order J-chain oligomers (Fig. S6*C*), showing much wider distribution in area than ERdj5 on the plot (Fig. S6*A*, middle). Concretely, whereas the areas of the ERdj5 molecules distribute mostly around 150 nm^2^ (Fig. S6*A*, left), a major portion of the J-chain particles show the area of less than 100 nm^2^ while a minor portion displaying the area of higher than 150 nm^2^ (Fig. S6*A*, middle). Such different distributions on the area *versus* circularity plots between the ERdj5 and J-chain particles enabled us to distinguish these two in the HS-AFM images of their mixtures with certainty (Fig. S6*A*, right).

Through repeated HS-AFM measurements, 106 J-chain oligomer molecules were observed, of which 62 were eventually disassembled or detached from the surface. During observations, some of the J-chain oligomers were transiently bound to multiple ERdj5 molecules at different sites until they were partially or fully disassembled (Fig. S7). We also observed that the same ERdj5 molecule attacked the same or similar sites of a J-chain oligomer repeatedly. In either case, ERdj5 molecules preferentially acted on the peripheral and fluctuating regions of J-chain oligomers rather than on their central core part. We measured the duration of ERdj5 binding to J-chain oligomers for two hundred ERdj5 molecule. Most ERdj5 molecules bound J-chain oligomers for <1.5 s per attack, irrespective of whether target J-chain oligomers were resolved or not by attack of the ERdj5 molecule (Fig. 6*D*). Thus, ERdj5 acts transiently on J-chain oligomers in most cases and subsequent J-chain disassembly *via* disulfide reduction seems likely to occur through repeated attacks by ERdj5 in a stochastic manner.

## Discussion

In this study, we successfully monitored the sequential reduction of J-chain oligomers catalyzed by the cooperation of ERdj5 and BiP. The results revealed that ERdj5 reduces J-chain oligomers in a stepwise manner *via* formation of trimeric and dimeric intermediates, while BiP alone resolves J-chain oligomers only partially in the presence of GSH. Importantly, BiP synergistically accelerated ERdj5-mediated J-chain reduction in an ATP-dependent manner (Fig. 1), and its functional interaction with the ERdj5 J-domain was essential for this acceleration (Fig. 3, *C* and *D*). BiP molecules activated by ERdj5 *via* its HPD motif may physically loosen aggregated parts of J-chain oligomers in advance and thereby increase the accessibility of ERdj5 and GSH to aberrant disulfide bonds. Excess BiP did not further enhance the ERdj5-catalyzed disulfide reduction (Fig. 2*E*), suggesting that the above events seem likely to be promoted in a 1:1 complex between ERdj5 and BiP. This mechanism appears to be consistent with a previously proposed model in which misfolded nonglycoproteins such as J-chain oligomers are first recognized by BiP and transferred to an ERdj5 molecule complexed with BiP in an ATP-dependent manner to accelerate disulfide reduction and ERAD of the substrates (10).

Our previous studies revealed that the C-terminal cluster of ERdj5 contains the primary reductase domains, and the highly dynamic nature of the N- and C-terminal clusters is key to efficient disulfide bond reduction of ERAD substrates (10, 15). The present biochemical experiments using purified proteins verified that the C-terminal Trx4 domain of ERdj5 plays a pivotal role in catalyzing disulfide reduction of J-chain oligomers and that limited cluster dynamics led to compromised J-chain reduction by ERdj5 (Fig. 5).

HS-AFM facilitated visualization of the actions of ERdj5 on J-chain oligomers at the single-molecule level. We found that ERdj5 molecules preferentially attacked the peripheral and flexible regions of J-chain oligomers rather than their central core parts. In most cases, ERdj5 bound the J-chain oligomers transiently and disassembled them by cleaving off small J-chain fragments. Thus, the enzyme is unlikely to bisect J-chain oligomers to nearly half-size fragments. These HS-AFM observations seem consistent with the results of J-chain reduction assays: J-chain oligomers appear to disassemble sequentially *via* the generation of trimeric and dimeric intermediates (Fig. 1).

It will be interesting to explore how many reductive reactions can occur during one binding or collision event. To calculate the frequency of disulfide bond reduction by one collision of ERdj5 with J-chain oligomers, the exact number of intermolecular disulfide bonds in a higher-order J-chain oligomer and the average interval time as well as the duration of the ERdj5 binding to the J-chain oligomers are necessary. Owing to the practical difficulties in these measurements, it is hard to precisely estimate how many disulfide bonds are reduced during one attack of an ERdj5 molecule against the J-chain oligomers. Nevertheless, our biochemical experiments showed that nearly 60% of the J-chain molecules were converted to the monomer after 60 min of the reaction at a 10:1 M ratio of J-chain to ERdj5 (Fig. 1*H*). Thus, the rate constant for the ERj5-catalyzed generation of the J-chain monomer is calculated to be 1 × 10^−1^ min^−1^, indicating a low efficiency of the reaction. Moreover, the HS-AFM analysis demonstrated that the release of small J-chain fragments from the J-chain oligomers occurred through repeated attacks of ERdj5, suggesting a low probability of disulfide reduction by one collision with ERdj5. These observations indicate the stochastic nature of the ERdj5-mediated J-chain reduction and disaggregation.

This study reconstituted the likely pathway along which misfolded nonglycoproteins are disaggregated by cooperation between ERdj5 and BiP in the ER. Accumulation of misfolded proteins stimulates the ER stress response and initiates the ERAD machinery. Indeed, both BiP and ERdj5 are stress-inducible (29, 30), which likely leads to further acceleration of ERAD pathways in cells. BiP is highly abundant in the ER and multiple BiP partners or activators are also present (31, 32, 33). It is therefore possible that misfolded protein aggregates are first handled by BiP and physically loosened to facilitate subsequent disulfide reduction and/or oligomer disassembly to smaller fragments. Consistently, preincubation with BiP greatly enhanced the ERdj5-mediated resolution of J-chain oligomers (Fig. 2, *G* and *J*). At the latter stages, BiP is believed to engage in substrate transfer from ERdj5 to a retrotranslocation channel upon ATP hydrolysis (9, 10). Meanwhile, nucleotide exchange factors such as SIL1 nucleotide exchange factor (SIL1) or glucose regulatory protein 170 likely promote substrate release from the ADP-bound form of BiP by facilitating the exchange of nucleoside diphosphate with nucleoside triphosphate (34, 35, 36). It will be interesting to determine whether these nucleotide exchange factors further accelerate the disulfide reduction cycle catalyzed by ERdj5 and BiP.

In conclusion, we confirmed the significant contribution of BiP in scavenging disulfide-linked protein oligomers in concert with ERdj5. The combinatorial approaches employed in this work deepen our understanding of the molecular mechanisms underlying the ERdj5-mediated ERAD pathway. Detailed analyses of the spatial distributions and dynamics of ERdj5, BiP, and other ERAD components using advanced microscopic imaging technologies will provide in-depth, functional views of how ERdj5 and BiP handle disulfide-linked oligomers for their efficient elimination in the ER.

## Experimental procedures

### Plasmid construction

ERdj5 mutants with an altered CXXC motif and ERdj5/S557P were constructed using a PrimeSTAR Mutagenesis Basal Kit (Takara Bio) and Gibson Assembly Master Mix (New England BioLabs) with appropriate sets of primers. The gene encoding human BiP was cloned into the PET15b vector containing an N-terminal His_6_-tag. The gene encoding human J-chain was cloned into the pCAGGS vector containing an N-terminal PA tag (37).

### Cell culture and transient transfection

HEK293T cells were cultured in Dulbecco's modified Eagle's medium (Nacalai Tesque) supplemented with 10% fetal bovine serum (Nichirei) and antibiotics (100 U/ml penicillin and 100 μg/ml streptomycin; Nacalai), in humidified air containing 5% CO_2_ at 37 °C. Transfection of plasmids encoding FLAG-tagged ERdj5 derivatives or PA-tagged J-chain was performed using PEI (Sigma-Aldrich) according to the protocol recommended by the manufacturer. Approximately 2 × 10^6^ cells were precultured in 15 cm dishes (Thermo Fisher Scientific) containing Dulbecco's modified Eagle's medium supplemented with 4% fetal bovine serum. At 24 h after plating, 20 μg of plasmid and 100 μg of 1 mg/ml PEI were mixed with 2 ml Opti-MEM (Gibco Service) for transfection, followed by culturing in humidified air containing 5% CO_2_ at 37 °C for 48 h.

### Protein expression and purification

Purification of recombinant ERdj5 was performed at 4 °C. Harvested cell pellets were suspended in lysis buffer (50 mM Tris–HCl pH 7.5, 150 mM NaCl, 10% v/v glycerol, 0.05% (w/v) Tween-20, protease inhibitor cocktail; Nacalai Tesque) using a potter-type homogenizer. Next, 1% (w/v) n-dodecyl-β-D-maltoside (Nacalai Tesque) was added to the cell lysate and samples were rotated. The mixture was centrifuged and the supernatant containing target protein was recovered and resuspended with anti-FLAG beads (Sigma-Aldrich) in buffer containing 50 mM Tris–HCl pH 7.5, 150 mM NaCl, and 10% (v/v) glycerol. After target protein binding, beads were washed with resuspension buffer, and protein was eluted with buffer containing 0.2 mg/ml FLAG peptide (Sigma-Aldrich). Samples at each stage were collected and analyzed by Western blotting with an anti-FLAG M2 antibody (F1804, Sigma-Aldrich, 1:20,000) to confirm the presence of FLAG-tagged ERdj5. All eluted fractions were mixed with 1-(3-sulfopropyl) pyridinium hydroxide inner salt (NDSB201; Tokyo Chemical Industry) to a final concentration of 1 M. Proteins were further purified by size-exclusion chromatography with a Superdex200 10/300 GL column (GE Healthcare) pre-equilibrated with buffer composed of 20 mM Hepes pH 7.5, 10% (w/v) glycerol, 0.05% (w/v) Tween-20, and 1 M NDSB-201. Eluted fractions containing ERdj5 were collected, concentrated, and stored at −80 °C. The final yield of ERdj5 WT from 4 g of cells was 0.53 mg.

The purification process for ERdj5 mutants with altered CXXC motifs, H63A, and S557P, was the same as for WT protein. The final yield of each ERdj5 mutant was as follows: ∼0.1 mg for ERdj5 with altered CXXC motifs from 1 g of cells; ∼0.3 mg for ERdj5 H63A from 4 g of cells; and ∼0.04 mg for ERdj5 S557P from 0.8 g of cells.

Purification of recombinant BiP was performed using *Escherichia coli* Rosetta (DE3) cells (Nippon Gene) carrying expression plasmids for BiP. The cells were cultured at 37 °C until the OD600 reached 0.6, at which point expression of recombinant protein was induced by adding 0.5 mM IPTG (Nacalai Tesque) and culturing was continued at 15 °C for 18 h. The cytosolic extract was mixed with nickel-charged affinity resin (nickel-nitrilotriacetic acid [Ni-NTA], agarose; QIAGEN) in Ni-NTA buffer (50 mM Tris–HCl pH 7.5, 500 mM NaCl, 2 mM MgCl_2_, 10% (v/v) glycerol, 0.2% Triton X-100) and 20 mM imidazole (Nacalai Tesque). Ni-NTA agarose beads that bound BiP were then washed with Ni-NTA buffer containing 3 mM ATP, and the protein was eluted with Ni-NTA buffer containing 0.5 M imidazole. Flow-through fractions containing BiP were further purified on a MonoQ column (GE Healthcare). As the final purification step, fractions containing target protein were applied to a Superdex 200 10/300 GL column (GE Healthcare) equilibrated in buffer containing 50 mM Tris–HCl pH 8, 150 mM NaCl, 10 mM MgCl_2_, and 10% (v/v) glycerol. Eluted fractions containing the target protein were collected and stored at −80 °C, and 0.4 mg of BiP was obtained from 2 l of culture.

Overexpression and purification of PA-J-chain were performed with HEK293T cells transfected with a plasmid overexpressing PA-J-chain using PEI and cultured for 48 h. Harvested cells were lysed in lysis buffer (50 mM Hepes pH 7, 150 mM NaCl, 1% NP-40, 0.1% SDS, 10% (v/v) glycerol, 10 mM NEM, and protease inhibitor cocktail) and homogenized by sonication. Lysates were clarified by centrifugation and precleared by incubation with Protein G Sepharose (GE Healthcare) for 1 h at 4 °C with rotation. Protein G Sepharose beads were removed by centrifugation and supernatants were incubated with anti-PA (NZ-27) antibody beads (FUJIFILM Wako Pure Chemical Corporation) equilibrated with J-chain wash buffer (50 mM Hepes-NaOH pH 7.0, 150 mM NaCl, 10% (v/v) glycerol, 1% v/v Nonidet P-40, 0.1% w/v SDS) for 2 h at 4 °C with gentle rotation. J-chain oligomers were eluted by buffer containing 0.2 mg/ml PA peptide (Sigma-Aldrich) and then further eluted with high-concentration magnesium solution. Samples at each stage were collected and analyzed by Western blotting with an anti-PA antibody (015-25951, FUJIFILM Wako, 1:20,000) to confirm the presence of PA-J-chain. Eluates containing PA-J-chain were desalted by chromatography on a PD-10 column (GE Healthcare) and concentrated by a membrane filter (Amicon Ultra; Merck Millipore) with a 100 kDa cut-off. A total of 0.23 mg PA-J-chain was obtained from 30 × 15 cm dishes.

Concentrations of all purified proteins were measured by the bicinchoninic acid method. All purified recombinant proteins were stored at −80 °C.

### ATPase activity measurement

ATPase activity of BiP was analyzed by quantifying the amount of phosphate group (Pi) released during hydrolysis of ATP by BiP. Quantification of free Pi was performed using an EnzChek Phosphate Assay Kit (Life Technologies corporation). Recombinant BiP or ERdj5 were incubated in assay buffer containing 20 mM Tris–HCl pH 7.5, 50 mM KCl, and 1.5 mM MgCl_2_. Protein at 2 μM was used for each reaction. The reaction was initiated by addition of 1 mM ATP at 37 °C. At various timepoints, 30 μl aliquots were removed and boiled at 100 °C for 5 min and then reacted with 2-amino-6-mercapto-7-methylpurine riboside and purine nucleoside phosphorylase for 30 min at 22 °C. In the presence of inorganic phosphate, 2-amino-6-mercapto-7-methylpurine riboside is converted enzymatically by purine nucleoside phosphorylase to ribose 1-phosphate and 2-amino-6-mercapto-7-methylpurine, resulting in a spectrophotometric shift of the absorbance peak from 330 nm to 360 nm. The increase in absorbance at 360 nm was monitored using a U-3310 spectrophotometer (Hitachi). The amount of free phosphate in the reaction solution was calculated from the increased absorbance, based on which the ATPase activity of BiP was estimated.

### J-chain oligomer reduction assay

Solutions containing proteins of interest, 2.5 mM GSH, and 1 mM ATP were prewarmed at 30 °C for 10 min and mixed well in assay buffer (50 mM Hepes pH 7, 0.05% (w/v) Tween-20, 10% (v/v) glycerol, 1 mM MgCl_2_, and 1 M NDSB-201). The reaction was started by mixing reagents with 5 μM J-chain oligomers and stopped by addition of 10 mM NEM on ice at different timepoints. Samples were separated by non-reducing SDS-PAGE and detected by CBB staining. Band intensities were quantified using Image Lab software (Bio-Rad Image Lab Software | Bio-Rad).

### High-speed AFM observation

Single-molecule images of ERdj5 and J-chain oligomers were acquired using a laboratory-built HS-AFM instrument (38, 39). Cantilevers (Olympus) were 6 to 7 μm long, 2 μm wide, and 90 nm thick. The spring constant was ∼0.1 Nm^−1^, and the resonant frequency and quality factor in water were 0.8 to 1 MHz and ∼2, respectively. The probe tip was grown on a cantilever by electron beam deposition and further sharpened by argon plasma etching (40). For AFM imaging, the free oscillation amplitude was ∼1.2 nm, and the set-point amplitude was ∼90% of the free oscillation amplitude. The estimated tapping force was <30 pN (41). For observation, a droplet (2 μl) containing 100 nM J-chain oligomer diluted in buffer containing 50 mM Tris–HCl pH 7, 150 mM NaCl, 5 mM MgCl_2_, and 2.5 mM GSH was loaded onto a cobalt-scattered mica surface. After 3 min of incubation, the surface was washed with buffer containing 50 mM Tris–HCl pH 7.0, 150 mM NaCl, 5 mM MgCl_2_, and 2.5 mM GSH to remove unbound J-chain molecules. For the HS-AFM measurement of J-chain alone, the J-chain–immobilized mica surface was incubated in the above buffer for 80 min before the acquisition of the HS-AFM images. For the measurement of the snapshots of the ERdj5-mediated J-chain reduction, ERdj5 (10 nM) diluted in the same buffer was added to the cantilever holder where the J-chain oligomer had been immobilized onto the mica surface (no preincubation with GSH), and the acquisition of the HS-AFM images was started immediately. Surface coating with Co^2+^ prevented the adherence of excess ERdj5 molecules. All AFM observations were performed at room temperature. Scan areas of 80 × 80 nm^2^ or 100 × 100 nm^2^ and a scan rate of 0.1 s/frame were used for standard observations. HS-AFM images were analyzed using the laboratory-built software Kodec4.4.7.39 (https://elifesciences.org/download/aHR0cHM6Ly9jZG4uZWxpZmVzY2llbmNlcy5vcmcvYXJ0aWNsZXMvMDQ4MDYvZWxpZmUtMDQ4MDYtY29kZTEtdjEuemlw/elife-04806-code1-v1.zip?_hash=NtPTIc%2Bx0E4UJPnezJJDPvvASgWDc8D8Xl8I5oRoEik%3D) (24). A low-pass filter was applied to images to remove noise (24, 38). For identification of observed particles, their areas and circularities were calculated. Circularity is a measure of how circular the outline of an observed molecule is defined by the equation 4πS/L2, where L and S are the contour length of the outline and the area surrounded by the outline, respectively. Thus, a circularity of 1 indicates a perfect circle, while values <1 indicate a more extended conformation.

## Data availability

All data described are contained within the article.

## Supporting information

This article contains supporting information.

## Conflict of interest

The authors declare that they have no conflicts of interest with the contents of this article.

## References

[1] Wedemeyer W.J., Welker E., Narayan M., Scheraga H.A. (2000). Disulfide bonds and protein folding. Biochemistry.

[2] Okumura M., Kadokura H., Inaba K. (2015). Structures and functions of protein disulfide isomerase family members involved in proteostasis in the endoplasmic reticulum. Free Radic. Biol. Med..

[3] Foster E.M., Dangla-Valls A., Lovestone S., Ribe E.M., Buckley N.J. (2019). Clusterin in Alzheimer’s disease: mechanisms, genetics, and lessons from other pathologies. Front. Neurosci..

[4] Tan J.M.M., Wong E.S.P., Lim K.-L. (2009). Protein misfolding and aggregation in Parkinson’s disease. Antioxid. Redox Signal..

[5] Back S.H., Kaufman R.J. (2012). Endoplasmic reticulum stress and type 2 diabetes. Annu. Rev. Biochem..

[6] Nishikawa S., Brodsky J.L., Nakatsukasa K. (2005). Roles of molecular chaperones in endoplasmic reticulum (ER) quality control and ER-associated degradation (ERAD). J. Biochem..

[7] Ruggiano A., Foresti O., Carvalho P. (2014). Quality control: ER-associated degradation: protein quality control and beyond. J. Cell Biol..

[8] Ellgaard L., Sevier C.S., Bulleid N.J. (2018). How are proteins reduced in the endoplasmic reticulum?. Trends Biochem. Sci..

[9] Ushioda R., Hoseki J., Araki K., Jansen G., Thomas D.Y., Nagata K. (2008). ERdj5 is required as a disulfide reductase for degradation of misfolded proteins in the ER. Science.

[10] Hagiwara M., Maegawa K., Suzuki M., Ushioda R., Araki K., Matsumoto Y. (2011). Structural Basis of an ERAD pathway mediated by the ER-resident protein disulfide reductase ERdj5. Mol. Cell.

[11] Oda Y., Hosokawa N., Wada I., Nagata K. (2003). EDEM as an acceptor of terminally misfolded glycoproteins released from Calnexin. Science.

[12] Molinari M., Calanca V., Galli C., Lucca P., Paganetti P. (2003). Role of EDEM in the release of misfolded glycoproteins from the Calnexin cycle. Science.

[13] Hoseki J., Ushioda R., Nagata K. (2010). Mechanism and components of endoplasmic reticulum-associated degradation. J. Biochem..

[14] Ushioda R., Hoseki J., Nagata K. (2013). Glycosylation-independent ERAD pathway serves as a backup system under ER stress. Mol. Biol. Cell.

[15] Maegawa K., Watanabe S., Noi K., Okumura M., Amagai Y., Inoue M. (2017). The highly dynamic nature of ERdj5 is key to efficient elimination of aberrant protein oligomers through ER-associated degradation. Structure.

[16] Shen Y., Meunier L., Hendershot L.M. (2002). Identification and characterization of a novel endoplasmic reticulum (ER) DnaJ homologue, which stimulates ATPase activity of BiP *in Vitro* and is induced by ER stress. J. Biol. Chem..

[17] Oka O.B.V., Pringle M.A., Schopp I.M., Braakman I., Bulleid N.J. (2013). ERdj5 is the ER reductase that catalyzes the removal of non-native disulfides and Correct folding of the LDL receptor. Mol. Cell.

[18] Inoue T., Dosey A., Herbstman J.F., Ravindran M.S., Skiniotis G., Tsai B. (2015). ERdj5 reductase cooperates with protein disulfide isomerase to promote simian virus 40 endoplasmic reticulum membrane translocation. J. Virol..

[19] Robinson P.J., Pringle M.A., Fleming B., Bulleid N.J. (2023). Distinct role of ERp57 and ERdj5 as a disulfide isomerase and reductase during ER protein folding. J. Cell Sci..

[20] Johansen F.E., Braathen R., Brandtzaeg P. (2000). Role of J chain in secretory immunoglobulin formation. Scand. J. Immunol..

[21] Hwang C., Sinskey A.J., Lodish H.F. (1992). Oxidized redox state of glutathione in the endoplasmic reticulum. Science.

[22] Montero D., Tachibana C., Rahr Winther J., Appenzeller-Herzog C. (2013). Intracellular glutathione pools are heterogeneously concentrated. Redox Biol..

[23] Lee A.S. (2005). The ER chaperone and signaling regulator GRP78/BiP as a monitor of endoplasmic reticulum stress. Methods.

[24] Kodera N., Yamamoto D., Ishikawa R., Ando T. (2010). Video imaging of walking myosin V by high-speed atomic force microscopy. Nature.

[25] Okumura M., Noi K., Inaba K. (2021). Visualization of structural dynamics of protein disulfide isomerase enzymes in catalysis of oxidative folding and reductive unfolding. Curr. Opin. Struct. Biol..

[26] Li Y., Wang G., Li N., Wang Y., Zhu Q., Chu H. (2020). Structural insights into immunoglobulin M. Science.

[27] Uchihashi T., Watanabe Y., Nakazaki Y., Yamasaki T., Watanabe H., Maruno T. (2018). Dynamic structural states of ClpB involved in its disaggregation function. Nat. Commun..

[28] Hirayama C., Machida K., Noi K., Murakawa T., Okumura M., Ogura T. (2021). Distinct roles and actions of protein disulfide isomerase family enzymes in catalysis of nascent-chain disulfide bond formation. iScience.

[29] Cunnea P.M., Miranda-Vizuete A., Bertoli G., Simmen T., Damdimopoulos A.E., Hermann S. (2003). ERdj5, an endoplasmic reticulum (ER)-resident protein containing DnaJ and thioredoxin domains, is expressed in secretory cells or following ER stress. J. Biol. Chem..

[30] Vitale M., Bakunts A., Orsi A., Lari F., Tadè L., Danieli A. (2019). Inadequate BiP availability defines endoplasmic reticulum stress. Elife.

[31] Pfaffenbach K.T., Lee A.S. (2011). The critical role of GRP78 in physiologic and pathologic stress. Curr. Opin. Cell Biol..

[32] Kohno K. (2010). Stress-sensing mechanisms in the unfolded protein response: similarities and differences between yeast and mammals. J. Biochem..

[33] Melo E.P., Konno T., Farace I., Awadelkareem M.A., Skov L.R., Teodoro F. (2022). Stress-induced protein disaggregation in the endoplasmic reticulum catalysed by BiP. Nat. Commun..

[34] Behnke J., Feige M.J., Hendershot L.M. (2015). BiP and its nucleotide exchange factors Grp170 and Sil1: mechanisms of action and biological functions. J. Mol. Biol..

[35] Rosam M., Krader D., Nickels C., Hochmair J., Back K.C., Agam G. (2018). Bap (Sil1) regulates the molecular chaperone BiP by coupling release of nucleotide and substrate. Nat. Struct. Mol. Biol..

[36] Yan M., Li J., Sha B. (2011). Structural analysis of the Sil1–Bip complex reveals the mechanism for Sil1 to function as a nucleotide-exchange factor. Biochem. J..

[37] Fujii Y., Kaneko M., Neyazaki M., Nogi T., Kato Y., Takagi J. (2014). PA tag: a versatile protein tagging system using a super high affinity antibody against a dodecapeptide derived from human podoplanin. Protein Expr. Purif..

[38] Noi K., Yamamoto D., Nishikori S., Arita-Morioka K., Kato T., Ando T. (2013). High-speed atomic force microscopic observation of ATP-dependent rotation of the AAA+ chaperone p97. Structure.

[39] Ando T., Kodera N., Takai E., Maruyama D., Saito K., Toda A. (2001). A high-speed atomic force microscope for studying biological macromolecules. Proc. Natl. Acad. Sci. U. S. A..

[40] Wendel M., Lorenz H., Kotthaus J.P. (1995). Sharpened electron beam deposited tips for high resolution atomic force microscope lithography and imaging. Appl. Phys. Lett..

[41] Rodríguez T.R., García R. (2003). Theory of Q control in atomic force microscopy. Appl. Phys. Lett..

