# Supplementary material for: Mechanistic characterization of disulfide bond reduction of an ERAD substrate mediated by cooperation between ERdj5 and BiP
Source: J Biol Chem. 2023 Sep 21;299(11):105274. doi: 10.1016/j.jbc.2023.105274 (PMC10591012; doi:10.1016/j.jbc.2023.105274)
Supplement: Supporting Figures S1–S7 [file mmc1.pdf]

Supporting Information for

Mechanistic characterization of disulfide bond reduction of an ERAD substrate mediated by cooperation between ERdj5 and BiP

Authors:

Xiaohan Cai[1),2)], Shogo Ito[1),2)], Kentaro Noi[3)], Michio Inoue[1)], Ryo Ushioda[4)], Yukinari Kato[5)], Kazuhiro Nagata[4)], Kenji Inaba[1),2),6),7)]*

[1)] Institute of Multidisciplinary Research for Advanced Materials, Tohoku University, Sendai, Miyagi 980-8577, Japan

[2)] Department of Molecular and Chemical Life Sciences, Graduate School of Life Sciences, Tohoku University, Sendai, Miyagi 980-8577, Japan

[3)] Department of Biotechnology and Life Science, Tokyo University of Agriculture and Technology, Koganei, Tokyo 184-8588, Japan

[4)] Faculty of Life Sciences, Kyoto Sangyo University, Kyoto 603-8555, Japan

[5)] Graduate School of Medicine, Tohoku University, Sendai 980-8575, Japan

[6)] Department of Chemistry, Graduate School of Science, Tohoku University, Sendai, Miyagi 980-8578, Japan

[7)] Core Research for Evolutional Science and Technology (CREST), Japan Agency for Medical Research and Development (AMED), Japan

Figure S1

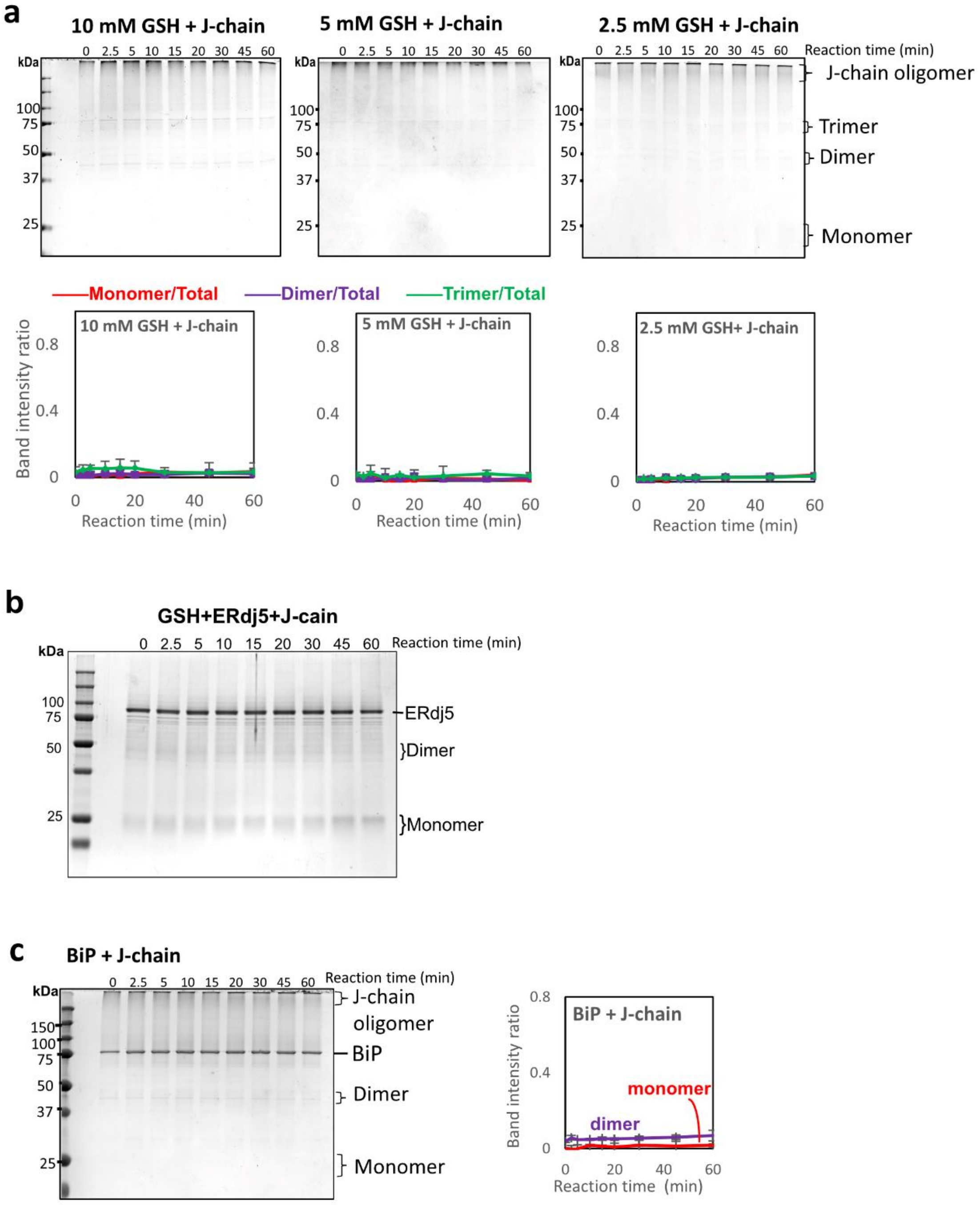

Figure S1. a. GSH concentration dependence of J-chain reduction. 5 μM J-chain oligomers were reacted with 10 mM, 5 mM, or 2.5 mM GSH for the indicated time at 30°C. Reaction solutions quenched with NEM at the indicated timepoints were subjected to non-reducing SDS-PAGE and stained with CBB G-250. Band intensity ratios of the J-chain monomer (red), dimer (magenta), and trimer (green) vs. total J-chain on the SDS gels are plotted as a function of reaction time (lower panels).

b. J-chain oligomers (5 μM) were reacted with 0.5 μM ERdj5 in the presence of 2.5 mM GSH for the indicated time at 30°C. After quenching with 10 mM NEM, the reaction solutions were subjected to reducing SDS-PAGE (1% β-mercaptoethanol). **c.** J-chain reduction mediated by BiP in the absence of GSH. J-chain oligomers (5 μM) were reacted with 0.5 μM BiP for the indicated time at 30°C. After quenching with 10 mM NEM, the reaction solutions were subjected to non-reducing SDS-PAGE and stained with CBB G-250. Band intensity ratios of J-chain monomer (red) and dimer (magenta) vs. total J-chain on the non-reducing SDS gel are plotted as a function of reaction time (right panel). Results are means ± SD of three independent experiments.

Figure S2

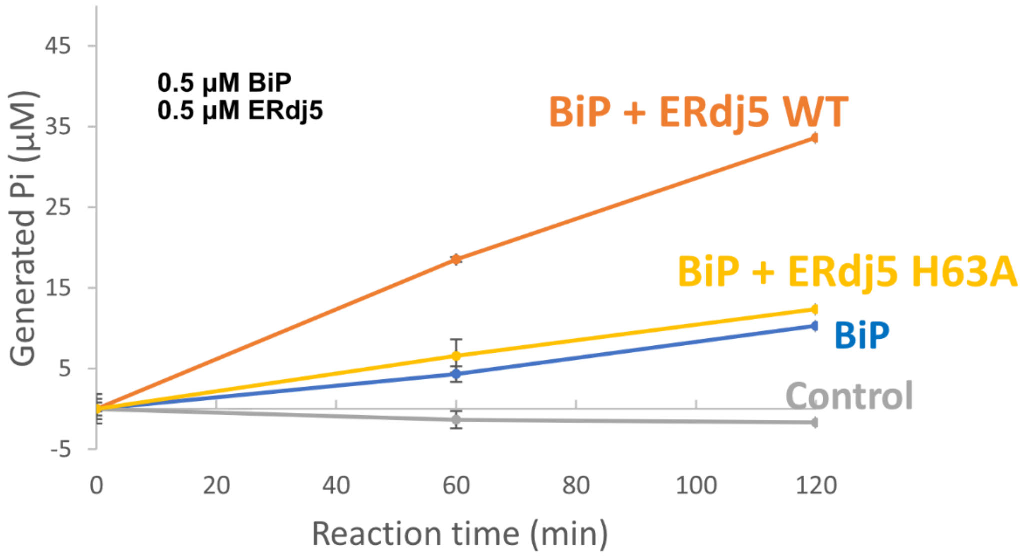

Figure S2. ATPase activity assay of BiP. 0.5 µM BiP and 1 mM ATP were reacted for 60 and 120 min in the presence or absence of ERdj5 WT/H63A, and phosphate released upon ATP hydrolysis was detected using an EnzChek Phosphate Assay Kit. Note that the ATPase activity of BiP was significantly increased by ERdj5 WT, but not by the ERdj5 H63A mutant. Results are means ± SD of three independent experiments.

Figure S3

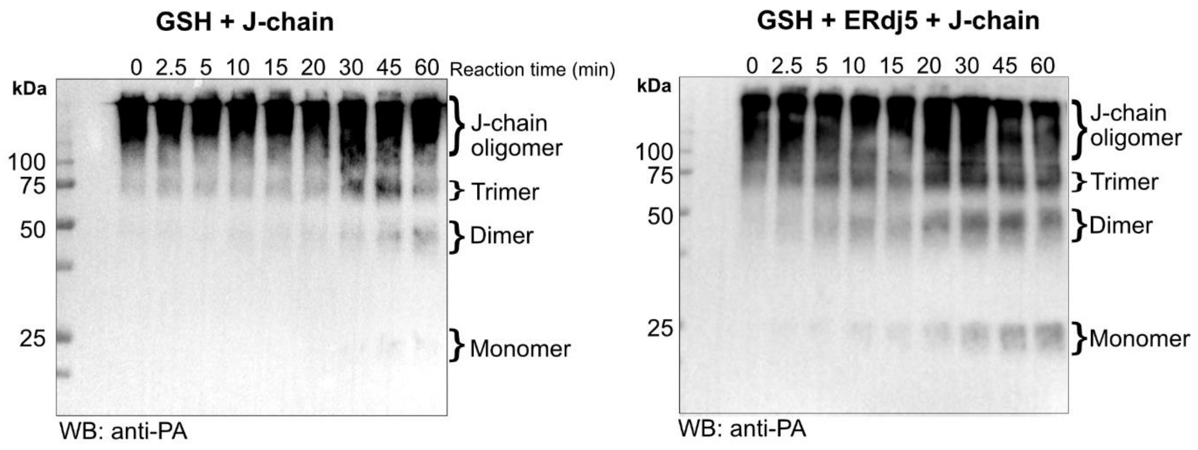

Figure S3. Western blotting analysis for the J-chain species generated during the reduction assay. 5 μM of the J-chain oligomers were reacted with 2.5 mM GSH in the absence (left) or presence (right) of 0.5 μM ERdj5 for the indicated time at 30°C. After quenching with 10 mM NEM, 0.5 μl of the reaction solutions were subjected to non-reducing SDS-PAGE. The PA-tagged J-chain species were visualized by western blotting with an anti-PA antibody (20,000 folds dilution, FUJIFILM Wako Pure Chemical Corporation).

Figure S4

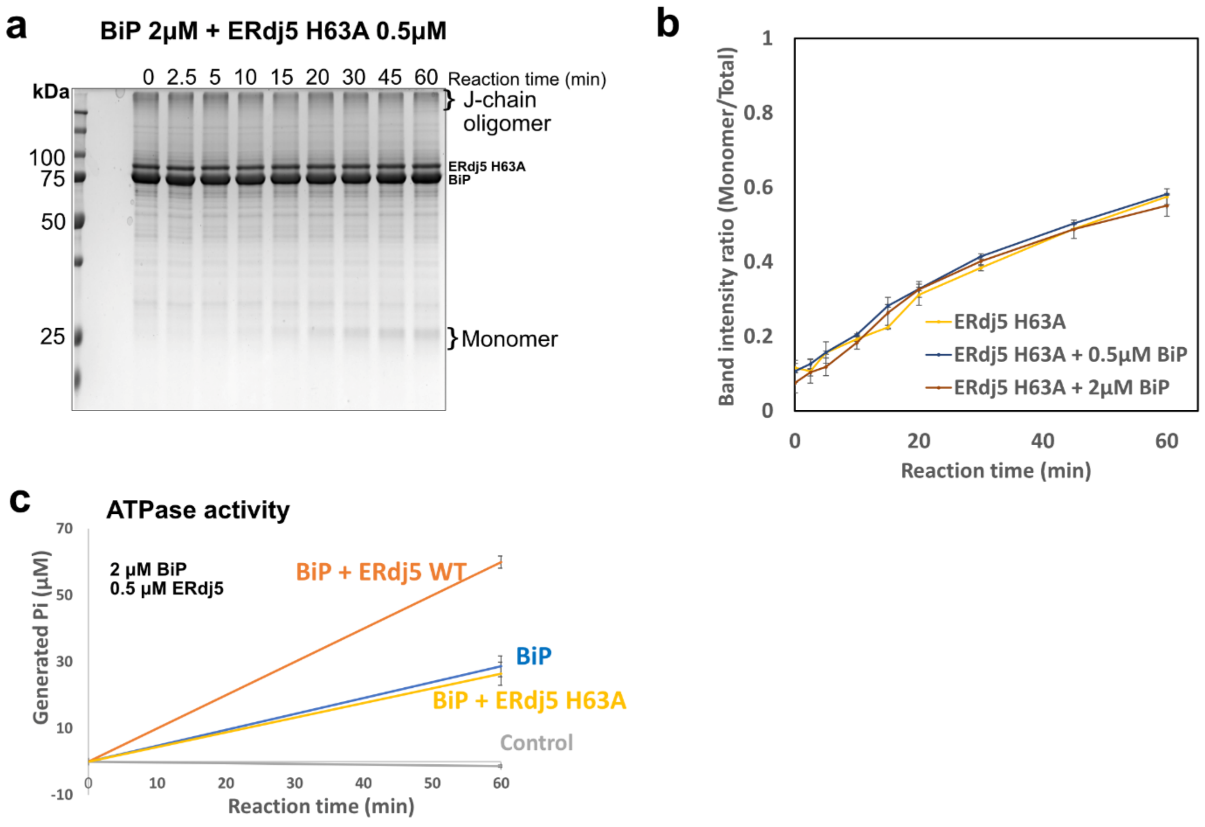

Figure S4. **a.** J-chain reduction mediated by 2 μM of BiP and 0.5 μM of the ERdj5 H63A mutant. 5 μM of the J-chain oligomers were reacted with 0.5 μM ERdj5 H63A, 2.5 mM GSH, and 1 mM ATP in the presence of 2 μM BiP for the indicated time at 30°C. After quenching with 10 mM NEM, the reaction solutions were subjected to non-reducing SDS-PAGE, followed by staining with CBB G-250. **b.** Band intensity ratio of J-chain monomer vs. total J-chain on the non-reducing SDS gel is plotted as a function of reaction time. Results are means ± SD of three independent experiments. **c.** ATPase activity assay of BiP. 2μM BiP was reacted with 1 mM ATP for 60 min, and phosphate released upon ATP hydrolysis was detected using an EnzChek Phosphate Assay Kit. Note that the ATPase activity of BiP was significantly enhanced by ERdj5, but not by the ERdj5 H63A mutant. Results are means ± SD of three independent experiments.

Figure S5

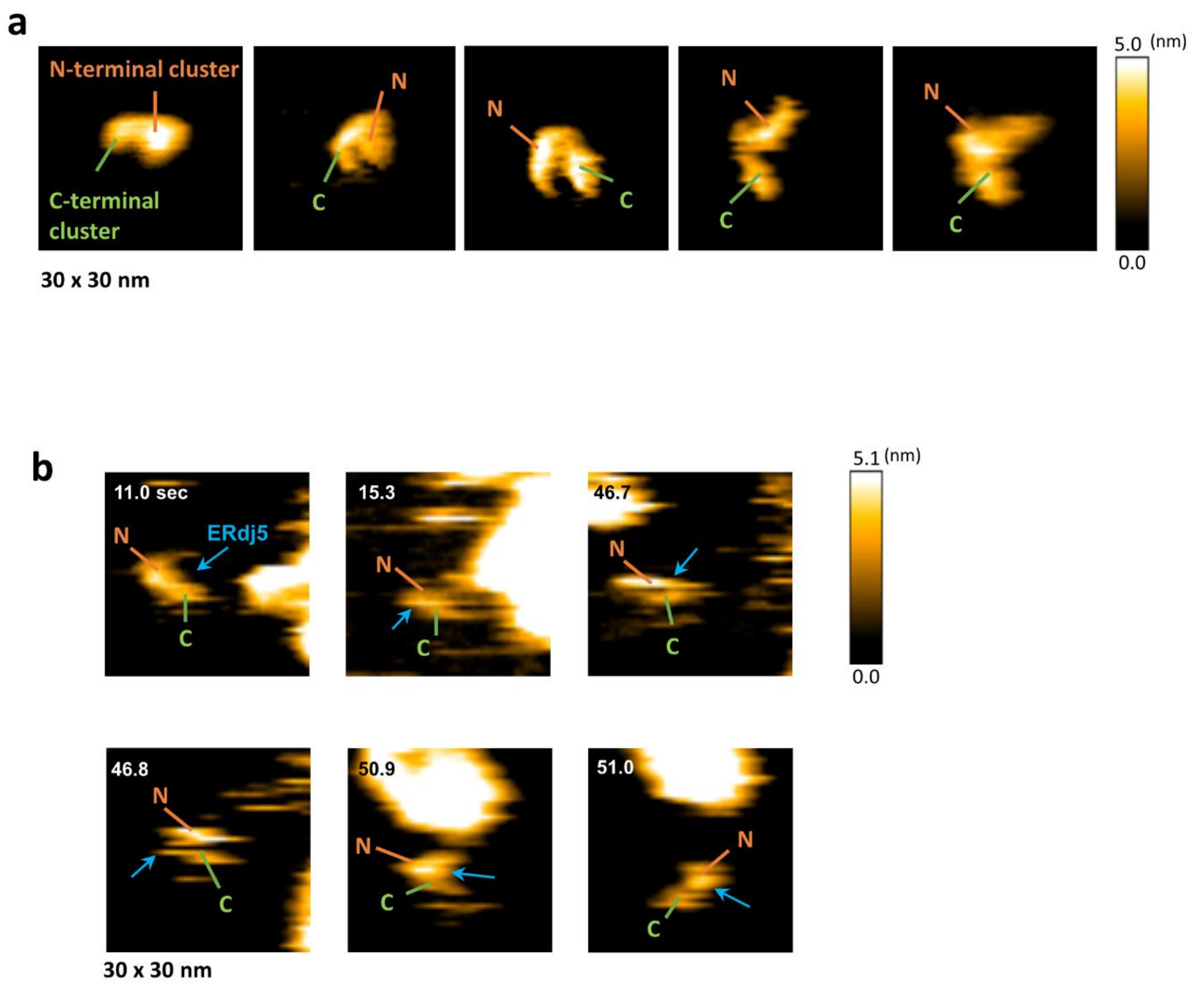

Figure S5. **a.** High-speed atomic force microscopy (HS-AFM) images of ERdj5 molecules. The labels "N" (orange) and "C" (green) in each image indicate the N- and C-terminal clusters of ERdj5, respectively. Note that the ERdj5 molecules adopt multiple conformations with different cluster orientations, consistent with previous observations (15, 25). **b.** Magnification of HS-AFM snapshots shown in Figure 6c. ERdj5 molecules are indicated by blue arrows.

Figure S6

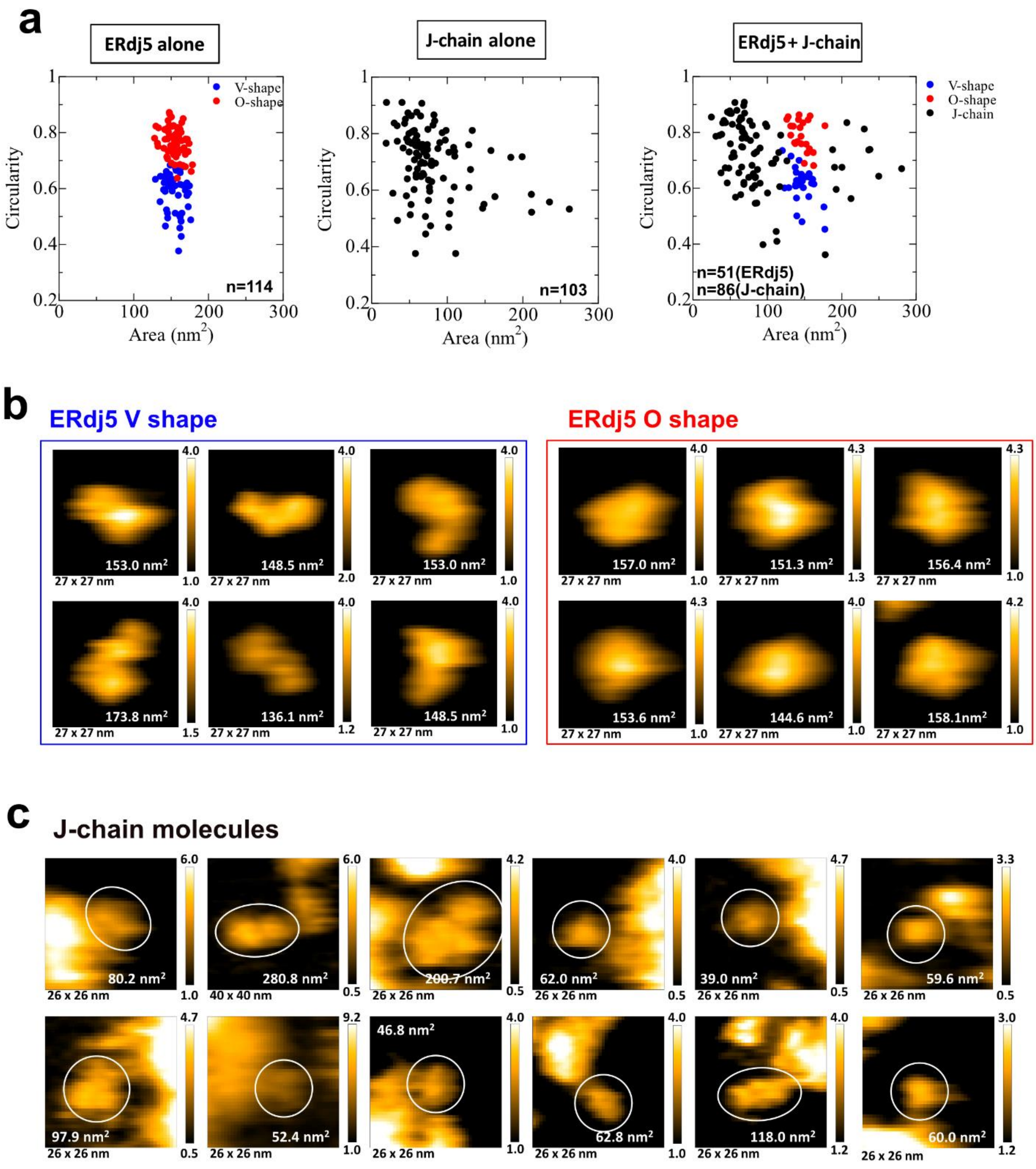

Figure S6. a. Two-dimensional scatter plots of the area versus circularity for the ERdj5 and J-chain particles in the presence of 2.5 mM GSH observed by HS-AFM. **b.** AFM images (scan area, 270 x 270Å) of ERdj5 molecules with the V-shape (left) and O-shape (right) conformations. **c**. Magnified AFM images (scan area, 260 x 260Å or 400 x 400 Å) of the J-chain fragments observed nearby the higher-order oligomers. The area values of the fragments are indicated by white letters in each image.

Figure S7

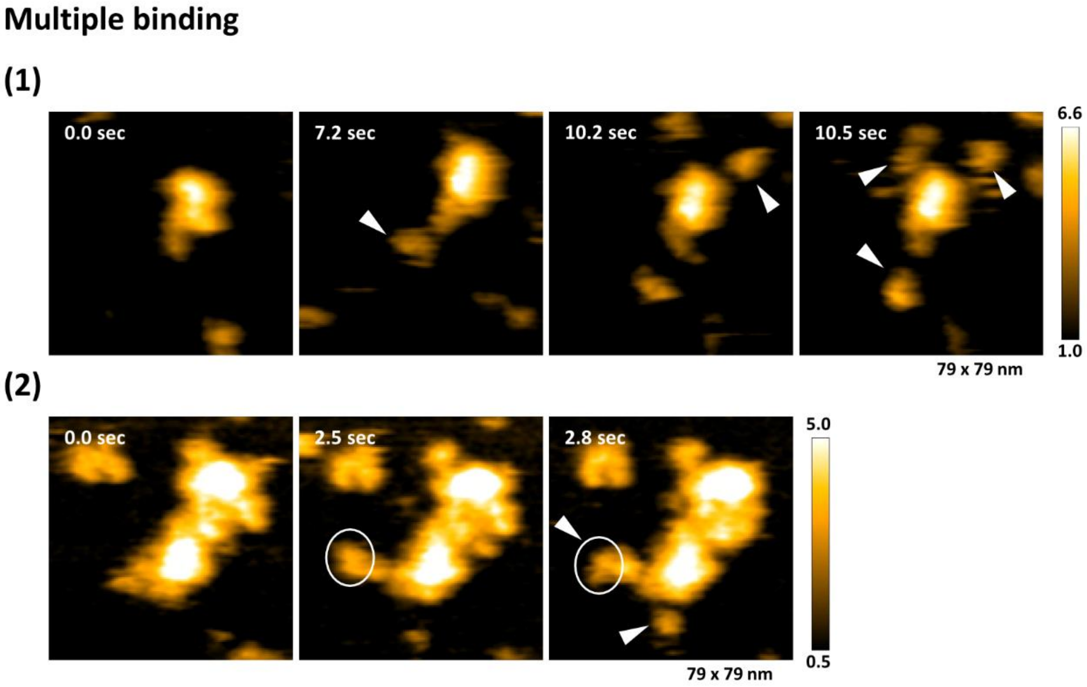

Figure S7. Transient binding of multiple ERdj5 molecules to one J-chain higher-order oligomer visualized by HS-AFM (scan area, 790 x 790Å). ERdj5 molecules are indicated by white arrows.
